# Creating the Trans Youth Research Network: A Collaborative Research Endeavor

**DOI:** 10.1089/trgh.2019.0024

**Published:** 2019-11-01

**Authors:** Johanna Olson-Kennedy, Yee-Ming Chan, Stephen Rosenthal, Marco A. Hidalgo, Diane Chen, Leslie Clark, Diane Ehrensaft, Amy Tishelman, Robert Garofalo

**Affiliations:** ^1^ Department of Pediatrics, Children's Hospital Los Angeles, Los Angeles California.; ^2^ Department of Pediatrics, University of Southern California, Los Angeles, California.; ^3^ Department of Pediatrics, Boston Children's Hospital, Boston, Massachusetts.; ^4^ Department of Pediatrics, Harvard Medical School, Boston, Massachusetts.; ^5^ Department of Pediatrics, Benioff Children's Hospital, San Francisco, California.; ^6^ Department of Pediatrics, University of California San Francisco, San Francisco, California.; ^7^ Department of Pediatrics, Lurie Children's Hospital, Chicago, Illinois.; ^8^ Department of Pediatrics, Northwestern Feinberg School of Medicine, Chicago, Illinois.

**Keywords:** gender affirming hormones, mental health of transgender youth, puberty suppression, transgender youth, Trans Youth Network

## Abstract

**Purpose:** This article outlines the process of establishing the Trans Youth Care Research Network, composed of four academic clinics providing care for transgender and gender-diverse (TGD) youth. The Network was formed to design and implement research studies to better understand physiologic and psychosocial outcomes of gender-affirming medical care among TGD youth.

**Methods:** Formed in response to both the Institute of Medicine's report recommendation for an increase of data concerning sexual and gender minority populations and a transgender-specific NIH program announcement, The Center for Transyouth Health and Development at Children's Hospital Los Angeles, the Gender Management Service at Boston Children's Hospital, the Child and Adolescent Gender Center Clinic at Benioff Children's Hospital in San Francisco, and the Gender and Sex Development Program at Lurie Children's Hospital of Chicago established a collaborative research network that subsequently designed a longitudinal observational study of TGD youth undergoing medical interventions to address gender dysphoria.

**Results:** Two cohorts, youth starting puberty blockers and youth starting gender-affirming hormones, are participating. Psychosocial measures that span multiple domains of mental and behavioral health are collected from youth and parents. Physiologic data are abstracted from patient's charts. Baseline and follow-up data of this large cohort will be disseminated through conferences, abstracts, posters, and articles.

**Conclusion:** Since initiation of funding in 2015, a total of 497 participants have been enrolled in TYC across the four sites; gonadotropin releasing hormone analogs (GnRHa) cohort youth (*n*=93), GnRHa cohort parents (*n*=93), and gender affirming hormone cohort youth (*n*=311). As the network moves toward data dissemination, its lessons learned have helped strengthen the current study, as well as inform future endeavors in this field.

## Introduction

### The psychosocial health of transgender and gender-diverse adolescents

Adolescence is a time of identity formation, during which youth are charged with exploration and growth, ultimately creating and pruning neural connections and pathways that are shaped by surroundings and experiences.^1^ Tremendous progress across educational, sexual, political, and other identity dimensions in this time of life forms the springboard from which adolescents will launch into adulthood.

Many health professionals have discarded the historical belief that transgender experience itself is pathological, replacing this ideology with an understanding that both gender dysphoria and the pervasive negative reactions from the environment are causal factors in the presenting mental health symptoms often evident among transgender and gender-diverse (TGD) individuals.

In Western culture, gender development occurs within a cultural context of cisgender normativity (i.e., an environment that assumes healthy development equates with having a gender identity that is aligned with one's designated sex at birth). Within this cultural context, where there are few-to-no road maps for TGD youth to follow in exploring their gender, TGD youth are challenged with an additional burden: discovery, disclosure, and stigma management of a gender different from what has been assumed based on their sex designated at birth. At odds with the social construct in which they are immersed, TGD youth may experience discomfort, stress, and anguish at both the societal/distal and individual/proximal levels.^2^

Distal stressors may include harassment, bullying, violence, loss of partner, family members, or community. These distal stressors are theorized to interact and bidirectionally influence individual/proximal stressors, such as internalized transphobia and negative expectations about the future, which in turn contribute to psychological distress. TGD youth experience high rates of anxiety and depression symptoms compared to cisgender adolescents, including sexually minoritized youth.^3^ Many carry diagnoses of attention deficit-hyperactivity disorder, obsessive–compulsive disorder, eating disorders, and others.^3–6^ Compared to cisgender peers, TGD youth engage in higher rates of self-harm, alcohol, and drug use, and more often have suicidal thoughts and attempts.^5–7^

In 2012, Spack et al. documented that among 97 youth, 20.6% had a history of self-injurious behavior, 9.3% a prior psychiatric hospitalization, and 9.3% a history of 1 or more suicide attempts.^4^ Results from a prospective study by Olson et al. reported that among 101 youth, depression scores were mild-to-moderate in 24% of participants and severe in 11%. Fifty-one percent reported contemplating suicide, and 30% had a history of at least one suicide attempt.^5^

Only a handful of studies have documented baseline well-being among TGD youth. Olson et al. found that socially-transitioned prepubescent gender-diverse children 12 years and younger were psychologically well adjusted compared to cisgender peers and siblings.^8^ Edwards-Leeper et al. found that a subset of adolescents seen in a transgender clinic seeking medical intervention appeared to be well adjusted based on standardized measures of mental health.^9^ Paramount to optimizing patient care is understanding the factors that play a role in the mental health of youth with gender dysphoria.

### The empirical knowledge gap

Over the past decade, a groundswell has occurred in the number of TGD adolescents seeking care at gender-specialized clinics throughout the United States and Western Europe.^4,10^ This growth is likely the result of a convergence of events, including the building of an accessible transgender community through the Internet, increased visibility of transgender narratives in media, and finally, increased availability of medical interventions. The broader visibility of TGD youth has opened the closet doors for many who would have previously remained nondisclosed about their gender for years, possibly even an entire lifetime.

Although transgender adults frequently report first recognizing their gender identities during childhood and adolescence, there is a consensus gap about the best approach to the care of youth with gender dysphoria. While a fair amount of extant literature supports the safety, positive impact, and importance of medical intervention for transgender adults,^11,12^ only a handful of studies focused on the impact of hormonal intervention on youth with gender dysphoria, particularly minors.^13–19^ As a result, there is lack of consensus among professionals around timing of initiation of medical interventions, as well as optimal dosing regimens.

The largest body of published data about transgender youth was derived from samples of Dutch youth. In 2006, the *Vrij Universiteit* Medical Center in Amsterdam disseminated the “Dutch Model” of care, outlining a protocol, including the use of puberty blockers to suppress endogenous puberty in youth with gender dysphoria starting at age 12, followed by the addition of estrogen or testosterone for induction of appropriate puberty starting at age 16.^15^ Follow-up data of 70 youth between the ages of 11 and 17 years initiating endogenous puberty suppression reported on mental health measures before initiation of blockers and then again before initiation of gender affirming hormones. Findings indicated a decrease in behavioral and emotional problems, a decrease in depressive symptoms, and improved general functioning for youth with gender dysphoria treated with puberty blockers.^16^

A follow-up study from the Dutch team examining the impact of puberty suppression followed by gender affirming hormones and gender reassignment surgery in 55 of the same sample (now in young adulthood) showed alleviation of gender dysphoria, steady improvement of psychological functioning, and a sense of “well-being” that was equivalent or superior to age-matched controls in the general population.^17^ While these findings are promising, the cohort is small, ethnically homogenous, and not necessarily generalizable to youth with gender dysphoria in the United States. To inform best clinical practices, the need for ongoing prospective and longitudinal data collection is clear.

### Forming the trans youth network

In 2011, the Institute of Medicine's (IOM) Report on the health of lesbian, gay, bisexual, and transgender populations called for increased investigation to better understand disparities within the lives of sexual and gender minoritized individuals, with specific discussion of transgender youth.^20^ Following the IOM report, the National Institutes of Health (NIH) released a program announcement aimed at better understanding the health of TGD populations in 2012, PA-12-111. For the first time in history, U.S. federal resources were allocated to understand and improve the lives of gender minoritized populations unrelated to HIV.

The Center for Transyouth Health and Development at Children's Hospital Los Angeles, the Gender Management Service at Boston Children's Hospital, the Child and Adolescent Gender Center Clinic at Benioff Children's Hospital in San Francisco, and the Gender and Sex Development Program at Lurie Children's Hospital of Chicago had been informally collaborating for several years before the release of the NIH program announcement. In response to the program announcement, these four centers agreed to work as a network and began formulating a study aimed at answering some of the most pressing questions about the care of TGD youth. All four sites, each possessing significant clinical experience providing medical care for youth with gender dysphoria, were well positioned to provide optimal environments for recruitment and retention of ethnically, geographically, and developmentally-diverse participants across a wide age span.

The four principal investigators (PIs) are two pediatric endocrinologists and two adolescent medicine subspecialists. All four sites have strong behavioral health professionals and psychologists on their teams, who serve as coinvestigators (Co-Is), working with PIs in conceptualizing research questions related to mental health outcomes, developing appropriate methodological strategies, choosing measures, and developing data analysis protocols for the planned study. The Network was formed with the purpose of designing and implementing research studies to better understand how medical interventions, social transition, youth resiliency, parental support, and affirmative environments affect the experiences of TGD youth.

### Trans Youth Care: The first network study

Recommendations from the World Professional Association of Transgender Health, the Endocrine Society, and cosponsoring organizations note that youth experiencing gender dysphoria in the early stages of puberty (Tanner stage 2–3) may benefit from the administration of gonadotropin releasing hormone analogs (GnRHa) to suppress further endogenous pubertal development.^21,22^ The use of GnRHa for youth beyond the early stages of puberty is also common, particularly in combination with gender affirming hormones. Youth experiencing gender dysphoria in their later or final stages of endogenous puberty may be appropriate for administration of gender affirming hormones to induce masculinizing or feminizing features.

The Trans Youth Care (TYC) Study was specifically designed to understand the impact of early medical treatment within two developmental cohorts: youth in early puberty with gender dysphoria seeking suppression of their endogenous puberty, and older adolescents and young adults with gender dysphoria seeking initiation of gender affirming hormones. Collecting data from each cohort over time is essential for understanding the long-term health and well-being of youth; hence, we used a longitudinal study design, expanding across 24 months of inquiry at 6-month intervals.

### Measurement domains

In a relatively emergent and fledgling field, prioritizing research questions is exceptionally challenging. Gathering as much data as possible must be balanced with the potential for overburdening participants with voluminous inquiry. This team was interested in the physiological outcomes of medical intervention, as well as understanding the social context of culture; family and peers; stigma and support; resilience; gender roles and expression; relationships and self-identity; traumatic experiences; mental health, including overlap of potentially co-occurring conditions such as addiction; obsessive–compulsive disorder; post-traumatic stress disorder; or developmental differences such as autism spectrum disorder.

### Physiologic measures

In light of existing data that underscore increased well-being among adolescents who initiate gender affirming hormones at younger ages,^18^ the first version of the Endocrine Society Guidelines (2009) recommended that gender affirming hormones be started in youth with gender dysphoria around the age of 16 years.^22^ The newest version of the Endocrine Society guidelines, published in 2017, acknowledges that there are compelling reasons to initiate gender affirming hormone treatment in youth with gender dysphoria younger than 16 years of age.^23^ Since this new recommendation, only a handful of studies have examined the physiologic impact of hormone use among younger adolescents.^13,14^

In contrast, the safety and impact of gender-affirming hormones used in adult TGD patients undergoing medical transition have been written about comprehensively and have demonstrated that hormones are relatively safe and result in a low risk profile for patients. The potential sequelae of gender-affirming hormone use in adults have been outlined, including risk for thrombotic events and changes in blood pressure, hemoglobin and hormone levels, and lipid profiles.^11^ While thrombotic events, hypertension, and lipid profile changes are sequelae exacerbated by aging (meaning fewer incidents will likely occur within our younger population), our team was interested in understanding whether youth had a similar or higher risk for these side effects based on introduction of exogenous hormones in adolescence.

While preliminary data concerning the impact of GnRHa have been published,^19,24^ their impact on bone health is one of the most important aspects to understand to optimize treatment protocols. In this study, physiologic data being collected include bone density, calcium intake, and weight-bearing exercise among participants to more fully understand the role of GnRHa within a wider context of bone health. A comprehensive list of metabolic, anthropometric, and bone health measures used is listed in Tables 1 and 2.

**Table 1.** Physiologic and Anthropometric Data—Early Pubertal Cohort (Puberty Suppression)

Time of completion: baseline, 6-, 12-, 18-, and 24-month follow-up periods	
Anthropometric data	Height, weight, blood pressure, BMI, Tanner stage
Laboratory results	Ultrasensitive luteinizing hormone, follicle stimulating hormone, estradiol, testosterone; calcium, alkaline phosphatase, serum phosphorus, vitamin D
QCT/DXA (blocker)	Read by male/female standards, radiologist read, endocrinologist read, QCT or DXA measurements

QCT/DXA, quantitative computed tomography/dual-energy x-ray absorptiometry.

**Table 2.** Physiologic and Anthropometric Data—Later Pubertal Cohort (Gender Affirming Hormones)

Time of completion: Baseline, 6-, 12-, 18-, and 24-month follow-up periods	
Anthropometric data	Height, weight, blood pressure, BMI, Tanner stage
Laboratory results	Fasting lipids and glucose, liver enzymes, electrolytes, prolactin level, hemoglobin, glycosylated hemoglobin, complete blood count, estradiol, and free and total testosterone
Gender affirming hormone cohort survey measures for subjects who were on blockers when they started puberty	
ACASI measures: time of completion	Baseline, 6-, 12-, 18-, and 24-month follow-up periods
**Construct**	**Measure**
Calcium intake	Daily calcium intake form
Weight bearing exercise	Physical activity questionnaire

ACASI, audio assisted computer survey instrument; BMI, body mass index.

### Psychosocial measures

The network hypothesized, from clinical experience, that many TGD youth carry historic and current mental health diagnoses due to the presence of symptoms that may diminish with gender-affirming medical intervention and an affirmative environment. To test this hypothesis, a protocol was developed to collect diagnostic information established before entry into care, along with diagnostic interviews conducted at baseline and at follow-up time points.

Initially, the team chose the Diagnostic Interview Schedule for Children (DISC), a validated, highly structured interview that assesses the presence of more than 30 mental health diagnoses.^25^ To decrease participant discomfort and potentially preserve data integrity, the team chose to replace the DISC with the Mini International Neuropsychiatric Interview for Children and Adolescents (MINI Kid and MINI), a shorter diagnostic tool that was not reported as distressful by participants.^26^ The team also opted to include symptom checklists to assess broadband internalizing and externalizing conditions, anxiety, depression, autism features, resilience, quality of life, body esteem, and suicidality. We hope that these symptom checklists will adequately discriminate nongender dysphoria-related, stand-alone mental health diagnoses (which we wouldn't necessarily expect to improve following medical intervention) versus “mental health” symptoms that are secondary to gender dysphoria that we would expect to improve following medical treatment. Comprehensive lists of the mental health measures are listed in Tables 3 and 4. The full protocol is outlined in a separate article.^27^

**Table 3.** Mental and Behavioral Health Measures—Early Pubertal Cohort (Puberty Suppression)

ACASI measures: time of completion	Baseline, 6-, 12-, 18-, and 24-month follow-up periods
**Construct**	**Measure**
Weight bearing exercise	Physical Activity Questionnaire
Demographics	Demographic questions for Blocker Cohort Youth
Depression	BDI-Y
Anxiety	Revised Children's Manifest Anxiety Scale: Second Edition (RCMAS-2—What I Think and Feel)
Quality of life	Pediatric Quality of Life Inventory—Child Report (PedsQL—CH)
Suicidality	Suicidal Ideation Scale
Body esteem	Body Esteem Scale
Social relationships	Emotional support/friendship/loneliness/perceived hostility/perceived rejection—NIH Toolbox
Self-harm	Questions about if and where participant has purposefully harmed themselves
Self-efficacy	Self-Efficacy (CAT 8–12/CAT 13–17)—NIH Toolbox
Perceived parent support	Parental Support Scale—Youth Version
Resiliency	Connor-Davidson Resilience Scale
Self-perception	Harter's Self-Perception Profiles for Adolescents & Children
Additional assessments: time of completion	Baseline, 12-, and 24-month follow-up periods
**Construct**	**Measure**
Mental health diagnoses	Mini International Neuropsychiatric Interview for Children and Adolescents—M.I.N.I. Kid^a^ (Modules: Major Depressive Episode/Manic Episode/Hypomanic Episode/Panic Disorder/Agoraphobia/Separation Anxiety Disorder/Social Anxiety Disorder (Social Phobia)/Specific Phobia/Obsessive–Compulsive Disorder/Post-traumatic Stress Disorder/Tourette's Disorder/ADHD/Conduct Disorder/Oppositional Defiant Disorder/Anorexia Nervosa/Bulimia Nervosa/Binge-Eating Disorder/Generalized Anxiety Disorder/Adjustment Disorders)
Additional assessments: time of completion	6-, 12-, 18-, and 24-Month follow-up periods
**Construct**	**Measure**
Side effects of GnRH agonists	Physical and emotional effects of hormone blocker use
Life Event Scale	Adolescent Life-Change Event Scale (Month 12 and 24 only)
Blocker Cohort—Parent Survey Measures	
ACASI Measures: time of completion	Baseline, 6-, 12-, 18-, and 24-month follow-up periods
**Construct**	**Measure**
Demographics	Demographic questions for Blocker Cohort Parents
Service utilization	Service Utilization Questions
Socioeconomic status	Socioeconomic Status Questions (for Adults)
Religiosity and spirituality	Modified Duke University Religion Index (DUREL)
Calcium intake	Daily Calcium Intake Form
Gender identity	Parent Report Gender Identity Questionnaire (GIQC)
Social transitioning	Social Transitioning Scale
Gender dysphoria	DSM 5—Chicago adapted
Quality of life	Pediatric Quality of Life Inventory—Parent Report (PedsQL—PC)
Suicide attempts	Suicidality Questions
Self-harm	Questions about if and where participant has purposefully harmed themselves
Parent distress/stress	Parenting Stress Index
Social relationships	Empathic Behaviors/Peer Rejection/Positive Peer Interactions/Social Withdrawal (Parent Report)—NIH Toolbox
Negative affect	Anger/Fear/Sadness (Parent Report)—NIH Toolbox
Psychological well-being	General Life Satisfaction/Positive Affect (Parent Report)—NIH Toolbox
Self-efficacy	Self-Efficacy (Parent Report)—NIH Toolbox
Perceived parent support	Parental Support Scale—Parent Version
Autism	Autism-Spectrum Quotient (AQ-10)—Child
Additional assessments: time of completion	Baseline, 12-, and 24-month follow-up periods
**Construct**	**Measure**
Depression/externalization	Child Behavior Checklist (CBCL)
DSM diagnoses^a^	Mini International Neuropsychiatric Interview for Children and Adolescents—M.I.N.I. Kid (Modules: Major Depressive Episode/Manic Episode/Hypomanic Episode/Panic Disorder/Agoraphobia/Separation Anxiety Disorder/Social Anxiety Disorder (Social Phobia)/Specific Phobia/Obsessive–Compulsive Disorder/Post-traumatic Stress Disorder/Tourette's Disorder/ADHD/Conduct Disorder/Oppositional Defiant Disorder/Anorexia Nervosa/Bulimia Nervosa/Binge-Eating Disorder/Generalized Anxiety Disorder/Adjustment Disorders)
Barriers to accessing care	Questions about perceived barriers to accessing a puberty blocker (Month 6 and 12 only)

^a^ Parent/caretaker participation optional.

ADHD, attention deficit-hyperactivity disorder; DSM, Diagnostic and Statistical Manual for Mental Disorders.

**Table 4.** Mental and Behavioral Health Measures—Later Pubertal Cohort (Gender Affirming Hormones)

ACASI Measures: time of completion	Baseline, 6-, 12-, 18-, and 24-month follow-up periods
**Construct**	**Measure**
Demographics	Demographic questions for Gender Affirming Hormone Cohort
Religiosity and spirituality	Modified Duke University Religion Index (DUREL)
Socioeconomic status	Socioeconomic Status Questions (for Adolescents & Young Adults)
Gender identity	Transgender Congruence Scale
Gender dysphoria	DSM 5—Chicago adapted
Service utilization	Dr. Olson-Kennedy's Service Utilization Questions
Depression	BDI-II
Anxiety	Revised Children's Manifest Anxiety Scale: Second Edition (RCMAS-2—What I Think and Feel)
Quality of life	Health-Related Quality of Life Scale (modified HIV QOL)
Suicidality	Suicidal Ideation Scale
Self-harm	Questions about if and where participant has purposefully harmed themselves
Body esteem	Body Esteem Scale
Body image	Body Image Scale
Social relationships	Emotional Support/Friendship/Loneliness/Perceived Hostility/Perceived Rejection—NIH Toolbox
Negative affect	Anger/Fear/Sadness—NIH Toolbox
Psychological well-being	General Life Satisfaction/Positive Affect—NIH Toolbox
Self-efficacy	Self-Efficacy (CAT 13–17)—NIH Toolbox
Perceived parent support	Parent Support Scale—Youth Version
Resiliency	Gender Minority Stress and Resilience Scale
	Connor-Davidson Resilience Scale
Sexual behavior	Sexual Risk Behavior Questions
STI history	STI Questions
Alcohol/drug use	Alcohol, Smoking, and Substance Involvement Screening Test (ASSIST)
Autism	Autism-Spectrum Quotient (AQ-10)—Adult
History of blocker experience	Questions to obtain history of participant's blocker experience
Additional assessments: time of completion	Baseline, 12-, and 24-month follow-up periods
**Construct**	**Measure**
Internalizing/externalizing	Youth Self-Report (YSR)
DSM diagnoses	Mini International Neuropsychiatric Interview—M.I.N.I. or M.I.N.I. Kid (Modules: Major Depressive Episode/Manic Episode/Hypomanic Episode/Panic Disorder/Agoraphobia/Social Anxiety Disorder (Social Phobia)/Obsessive–Compulsive Disorder/Post-traumatic Stress Disorder/Anorexia Nervosa/Bulimia Nervosa/Binge–Eating Disorder/Generalized Anxiety Disorder/Antisocial Personality Disorder)^a^
Additional assessments: time of completion	6-, 12-, 18-, and 24-Month follow-up periods
**Construct**	**Measure**
Side effects of hormone use	Physical and emotional effects of hormone use for the following hormone treatments: testosterone, progesterone, estrogen, spironolactone, and/or other hormone blockers
Physical characteristics	Menstruation (first and last menstrual period); history of chest binding and male chest reconstruction procedures; interest in gender affirming surgeries (for transmasculine participants *only*)
Chest dysphoria	Chest Dysphoria Scale (for transmasculine participants *only*)
Life Event Scale	Adolescent Life Change Event Scale (Month 12 and 24 only)
Gender dysphoria	Utrecht Gender Dysphoria Scale (UGDS) (Month 24 only)
	Gender Identity/Gender Dysphoria Questionnaire Adolescents & Adults (Month 24 only)
Participant feedback	Questions to obtain participant perceptions about their experience of participating in the study and feedback about some items of measures used (Month 24 only)
Barriers to accessing care	Questions about perceived barriers to accessing gender affirming hormones (Month 24 only)

^a^ The M.I.N.I. Kid will be utilized with participants aged 16 and under at the Baseline Visit, and the M.I.N.I. will be utilized with participants17 and older at the Baseline Visit. Refer to table of “Blocker Cohort—Youth Survey Measures” for modules included in M.I.N.I. Kid interview.

### Challenges of existing measures

Existing measures of depression, anxiety, coping, resilience, suicidality, and self-harm are not specifically validated for TGD youth. In addition, there are no recommendations for scoring those tools (e.g., CBCL, YSR) that are normed by gender.^28^ In those cases when we utilize scales scored by gender, we are choosing to score participants according to both male and female parameters as suggested by some.^28^ The existing measures of gender dysphoria are inadequate on multiple levels.

The Utrecht Gender Dysphoria Scale (UGDS) is a short survey with questions regarding the distress persons feel when confronted in daily life with the fact that the gender they are living in that aligns with the sex designated at birth is incongruent with their own inner feelings about their gender.^29^ The UGDS is static in nature, capturing elements of an individual's physical experience and not likely to capture change over time. For example, in the scale designed to be administered to those designated female at birth, the item, “I hate having a menstrual cycle,” will likely continue to receive affirmative responses from those who identify as male or a gender other than female, even if amenorrhea is achieved through medical intervention.

UGDS items that inquire about sexuality such as, “I like to behave sexually like a girl,” posit significant generalities about sexual behavior and assume that such behaviors are inherently gendered. The UGDS is binary in nature, thereby rendering it inadequate for capturing information about youth with nonbinary gender identities (i.e., neither male nor female, but an intermediate gender or some combination of both).

The necessity for an improved measure to capture the nuanced elements of gender dysphoria and its potential for intensification or mitigation over time has been highlighted by our transgender team members who have been on the front line with youth participating in the study. After significant deliberation, we chose to include the UGDS in this study, in the hopes that we might demonstrate its limitations in capturing the dynamic experience of youth with gender dysphoria. For similar reasons, we also included The Gender Identity/Gender Dysphoria Questionnaire for Adolescents and Adults (GIGDQAA).^30^

Over the course of the past 2 years, there were concerns articulated by participants regarding distress they experience when confronted with some of the items from both of these instruments. A decision was made to remove the UGDS and GIGDQAA from the participant assessments, except for those participants completing a substudy to obtain participant feedback regarding items included in those two scales. Our team felt that the Transgender Congruence Scale^31^ and the Gender Minority Stress and Resilience Scale^32^ are likely the best existing measures to collect information about both distal and proximal drivers of gender dysphoria.

### Models of care

While guidelines exist that provide recommendations for treatment timelines and protocols, a lack of treatment outcome data yields significant room for interpretation of such recommendations. All four gender clinics include medical professionals from the subspecialties of either endocrinology or adolescent medicine, nursing, psychiatry, psychology, and social work.

The four sites in this study practice differently with respect to psychological assessment of youth before medical intervention, intake procedures, types of puberty blocking medications, hormones used for feminization, timing of initiation of medical interventions, dosing regimens, as well as medication monitoring and adjustment. No evidence outlining the comparative benefits or risks among different practice models exists, and considerable debate persists within professional communities about best practices.

The differences among sites provide an opportunity to study differential effects of these care models; conversely, the differences in practices may result in variability of results that can pose challenges in drawing general conclusions about elements of care shared between all sites.

### Current enrollment demographics

Since initiation of funding in 2015, a total of 497 participants have been enrolled in TYC across the four sites; GnRHa cohort youth (*n*=93), GnRHa cohort parents (*n*=93), and GAH cohort youth (*n*=311).

### Lessons learned to date

Over the past two decades, community-based participatory research (CBPR) has increasingly been considered an important strategy for eliminating racial and ethnic health disparities by engaging community members as partners in research design, collaborative discourse about knowledge, and in intervention development and health policy-making.^33^ The employment of a CBPR model in studies involving transgender populations is also likely to benefit from such an approach. This first investigation did not use CBPR methods but prioritized the hiring of transgender research professionals who informed the development and modification of research protocols.

The voices of our transgender team members cannot represent the entire community, but they bring unique perspective and input to the process of identifying appropriate measures, decision-making around analysis and interpretation of measures that are scored based on gender, and provide important critiques regarding the value of certain aspects of the study to the community itself. Ongoing meetings with input from transgender team members have informed the network's consideration of CBPR methods to use as study aims evolve over the course of investigating these two developmental cohorts of youth.

## Conclusion

As our network moves toward data dissemination, our lessons learned have helped strengthen our current study, as well as inform future endeavors. Perhaps most important is the greater understanding that transgender identity and gender dysphoria are complicated, multifactorial, and extraordinarily nuanced human experiences.

While some common features exist among transgender youth, the developmental trajectories and needs are unique to each individual and are impacted by innumerable factors, including physiology, psychology, temperament, genetics, familial structure, sociocultural factors, geography, religion, ideology, exposure, politics, and others. In addition, provider-related factors, including personal beliefs, religion, culture, ideology, exposure, and knowledge, will also lead to differences in how care is approached and practiced. Finally, commitment to community engagement for the ongoing investigation will infuse the work with the critical perspective of the community the study seeks ultimately to serve.

## Abbreviations Used

CBPR: community-based participatory research
DISC: Diagnostic Interview Schedule for Children
GIGDQAA: Gender Identity/Gender Dysphoria Questionnaire for Adolescents and Adults
GnRHa: gonadotropin releasing hormone analogs
IOM: Institute of Medicine
NIH: National Institutes of Health
PI: principal investigator
TGD: transgender and gender-diverse
TYC: Trans Youth Care
UGDS: Utrecht Gender Dysphoria Scale
